# Comparing the corneal temperature of dry eyes with that of normal eyes via high-resolution infrared thermography

**DOI:** 10.3389/fmed.2024.1526165

**Published:** 2025-01-07

**Authors:** Chunbo Wu, Yuanshen Huang, Banglian Xu, Baicheng Li, Songlin Zhuang, Guofan Cao, Yan Hu, Zhensheng Gu

**Affiliations:** ^1^School of Optical Electrical and Computer Engineering, University of Shanghai for Science and Technology, Shanghai, China; ^2^Affiliated Eye Hospital of Nanjing Medical University, Nanjing, China; ^3^Department of Ophthalmology, Xinhua Hospital, School of Medicine, Shanghai Jiao Tong University, Shanghai, China

**Keywords:** dry eye disease, DED, ocular surface temperature, corneal temperature, ocular thermography, high-resolution infrared thermography

## Abstract

**Purpose:**

This study compares the corneal temperature in dry eyes with normal eyes via high-resolution infrared thermography.

**Methods:**

A total of 86 participants were enrolled, with 40 and 46 participants in the dry eye disease (DED) and control groups, respectively. All participants underwent non-invasive breakup time (NIBUT) measurement, an Ocular Surface Disease Index (OSDI) questionnaire and ocular thermography.

**Results:**

In the DED group, the mean initial central corneal temperature (initial CCT) is 33.25 ± 0.66°C, the tenth-second central corneal temperature (10s-CCT) is 32.47 ± 0.84°C, and the mean change in central corneal temperature measured within 10 s (change in CCT within 10 s) is 0.78 ± 0.30°C. For the controls, the initial CCT, 10s-CCT, and change in CCT within 10 s are 33.14 ± 1.02°C, 32.90 ± 0.99°C, and 0.23 ± 0.20°C, respectively. Except for the initial CCT (*p* = 0.549), significant differences are observed in the 10s-CCT (*p* = 0.034) and the change in CCT within 10 s (*p* < 0.001) between the two groups. The standard deviation of the temperature values within the region of interest (SD of TVs within ROI) on the central cornea is calculated to compare the uniformity of corneal temperature. In the DED group, the mean standard deviation of the initial temperature values within the region of interest (SD of initial TVs within ROI) is similar to that in the control group (0.23 ± 0.07°C vs. 0.22 ± 0.05°C, *p* = 0.926). In contrast, the mean standard deviation of the tenth-second temperature values within the region of interest (SD of 10s-TVs within ROI) in the DED group is greater than that in the control group, and there is a significant difference (0.44 ± 0.20°C vs. 0.35 ± 0.15°C, *p* = 0.016). In the DED group, the mean change in CCT within 3 s after tear film break-up is significantly greater than that before tear film break-up (0.19 ± 0.08°C vs. 0.10 ± 0.10°C, *p* < 0.001).

**Conclusion:**

As the time with eyes open increases, dry eyes present a significantly faster decrease in central corneal temperature (CCT) and a significantly worse uniformity of corneal temperature compared with normal eyes.

## 1 Introduction

Dry eye disease (DED) is a common ophthalmic disorder that can lead to ocular discomfort, reduced visual acuity, and a decline in both visual function and overall quality of life (1–3). It has been reported that 25% of patients attending ophthalmic clinics exhibit symptoms associated with dry eye syndrome (4, 5). According to the latest research updates, the Tear Film and Ocular Surface Society (TFOS) Dry Eye Workshop (DEWS) II has updated the definition of DED to describe it as a multifactorial disease affecting the ocular surface. This condition is marked by a disruption in the tear film’s homeostasis and is associated with ocular symptoms. Key factors in its development include tear film instability and hyperosmolarity, inflammation and damage to the ocular surface, as well as neurosensory abnormalities (6).

The impaired stability of the tear film is a key diagnostic criterion for identifying abnormalities in the tear film. The TFOS DEWS II Diagnostic Methodology report outlines various methods for assessing tear film stability, including fluorescein breakup time (FBUT) (7, 8), non-invasive tear breakup time (NIBUT) (9, 10), thermography (11, 12), osmolarity variability (13, 14), and tear evaporation rate (15, 16), among others. Among these ways, the thermography has facilitated assessments of ocular surface temperature (OST) through a non-invasive methodology (11, 12, 17–19). It is widely recognized that OST is associated primarily with the tear film (20). Studies have shown that OST and FBUT can be measured simultaneously (21, 22), revealing that the areas experiencing cooling and breakup of the ocular surface coincide (22). There is a direct correlation between FBUT and ocular surface cooling, suggesting that localized evaporation increases contribute to the thinning and breakup of the tear film (21). Furthermore, technological progress in instrumentation has enhanced the ability to measure OST with greater accuracy, resolution, and speed.

Recent and historical research has highlighted the distinctions in OST between normal eyes and those affected by DED (11, 12, 23–25). However, there has been limited analysis of the details of OST. The main reason for this is that previous infrared thermography devices had low pixel resolution, making it difficult to capture detailed differences in OST. Nevertheless, assessing these detailed differences in OST is also important for evaluating the differences between dry eyes and normal eyes (12, 26). Therefore, we compared the central corneal temperature (CCT) and uniformity of the corneal temperature between dry eyes and normal eyes via high-resolution infrared thermography to further explore the role of infrared thermography in the diagnosis of dry eyes.

## 2 Materials and methods

### 2.1 Participants

All procedures conducted in this study were in accordance with the Declaration of Helsinki. Approval for the research was granted by the Ethics Committee of Xin Hua Hospital Affiliated to Shanghai Jiao Tong University School of Medicine (Approval No. XHEC-D-2023-047). Informed consent was obtained from all participants before their enrolment in the study. After providing informed consent, a total of 86 consecutive Asian individuals, comprising 40 individuals with DED and 46 individuals with normal ocular conditions, participated in this observational study. The diagnosis of DED was established based on the criteria established by the Tear Film and Ocular Surface Society (TFOS) Dry Eye Workshop (DEWS) II Diagnostic Methodology Subcommittee (27).

The exclusion criteria for the study included individuals with a history of ocular surgery or trauma, those under the age of 18, patients who have undergone chalazion excision, individuals experiencing acute inflammation, and those with a documented history of blepharal or periorbital skin conditions or allergies within the past month. Additionally, participants with severe dry eyes accompanied by corneal epithelial defects, limbic keratitis, pterygium, corneal neovascularization, glaucoma, rheumatic autoimmune diseases, or a history of herpes zoster infection were excluded, as well as pregnant individuals, contact lens users, and those with a history of antihistamine or antidepressant medication use.

### 2.2 Non-invasive breakup time

Non-invasive breakup time (NIBUT) was evaluated noninvasively via the Keratograph 5 M (Oculus, Germany) topographer. Three consecutive measurements were obtained, and the median value was used for the diagnosis of DED (28).

### 2.3 Ocular surface disease index

The Ocular Surface Disease Index (OSDI) was used to evaluate and quantify the symptoms associated with DED. The questionnaire comprises 12 items, which can be aggregated into a score that spans from 0, indicating the absence of symptoms, to 100, indicating severe symptoms.

### 2.4 Ocular thermography

Thermal imaging and video recordings were obtained via an infrared thermal camera (Xcore LT640, IRay Tech Co., Ltd., China) operating within the long-wave spectrum (8–14 μm). The camera is characterized by a frame rate of 30 Hz and a spatial resolution of 640 × 512 pixels, with each pixel measuring 14 × 14 μm, and an accuracy of measurement of ±2%. Before the initiation of testing, the infrared thermal camera was preheated for 20 min, employing automatic non-uniformity correction with a correction interval of 1 min. Furthermore, each participant was instructed to rest for 10 min in the examination room. The environmental conditions, specifically temperature and humidity, were monitored and regulated to maintain a consistent range of 22 ± 1°C and 40 ± 5%, respectively. Measurements were conducted during standard hospital operating hours, specifically from 8 AM to 11 PM. The measurement protocol included the following steps: 1. The participants were directed to maintain a stable head position while their chin and forehead rested and their gaze was focused on a predetermined focal point; 2. The infrared thermal camera recorded the temperature of each participant’s forehead; 3. The participants were instructed to open their eyes, blink, and subsequently maintain their eyes in an open position for 10 s, during which the infrared thermal camera captured a video of the right eye.

### 2.5 Image acquisition and analysis methods

**Figure 1:**
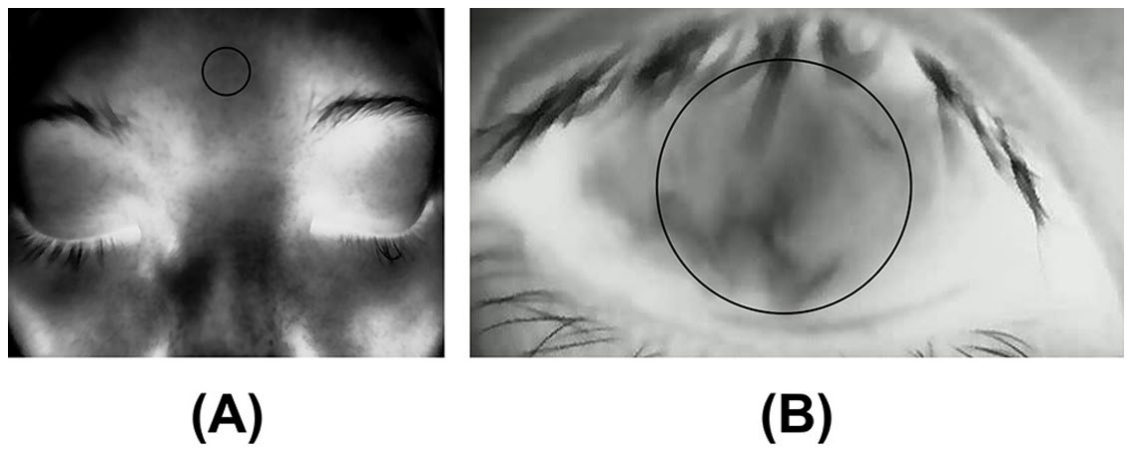
**(A)** Forehead temperature: The temperature was measured at the forehead; **(B)** Central corneal temperature (CCT): The average temperature was measured within ROI (8mmφ) on the central cornea.

Image post-processing was conducted via custom-developed MATLAB scripts (MathWorks, Inc.). The average temperature readings were obtained from two specific locations: the forehead (Figure 1A) and the ocular surface (Figure 1B). The designated region of interest (ROI) on the ocular surface corresponds to the central cornea, which has a diameter of 8 mm (29, 30).

The measurement of ROI is semiautomatic. This infrared thermal camera allows for customizable ROI, providing the average temperature within the region. Therefore, we predefined a circular ROI with a diameter of 160 pixels on the display interface of the infrared thermal camera. After focusing, this area corresponded to a circular region of approximately 8 mm in diameter on the central cornea. Data collection commenced once the eye was adequately open, ensuring that the upper eyelid was no longer elevated, thereby allowing the ROI on the ocular surface to be unobstructed by the eyelid or eyelashes. This approach facilitated the acquisition of the earliest possible measurements of central corneal temperature (CCT).

Upon achieving adequate eyelid elevation, the ROI was positioned at the center of the ocular surface, ensuring that there was no obstruction from the eyelids or eyelashes, to assess variations in the CCT. This initial measurement was designated 0 s. A total of 11 infrared thermal images of the ocular surface were subsequently extracted from an infrared thermal video at one-second intervals over 10 s for each eye. At each time point, the mean temperature of the central cornea was calculated and recorded. As a result, we were able to analyse the alterations in infrared thermal images of CCTs within the 10 s following the opening of the eye.

The thermogram was stored in irg format. According to the file in irg format, we calculated the standard deviation of the temperature values within the ROI (SD of TVs within ROI) to compare the uniformity of corneal temperature between the DED and control groups (29).

### 2.6 Statistical methods

Categorical variables were analysed through frequency rates and percentages, whereas continuous variables that followed a normal distribution were summarized using means and standard deviations. To assess the sex ratio between the DED group and the control group, a chi-square test was employed. Unpaired t-tests were used to evaluate differences in age, forehead temperature, non-invasive breakup time (NIBUT), Ocular Surface Disease Index (OSDI), initial central corneal temperature (initial CCT), tenth-second central corneal temperature (10s-CCT), change in central corneal temperature measured within 10 s (change in CCT within 10 s), standard deviation of the initial temperature values within the ROI (SD of initial TVs within ROI), and standard deviation of the tenth-second temperature values within the ROI (SD of 10s-TVs within ROI) between the DED and control groups. Unpaired t-tests were also used to evaluate the differences in initial CCT, 10s-CCT and change in CCT within 10 s between males and females in the only DED group, the only control group, and both groups. We used paired t-tests to evaluate the change in CCT within 1 s before and after tear film break-up, the change in CCT within 2 s before and after tear film break-up, and the change in CCT within 3 s before and after tear film break-up. All the statistical analyses were performed via the Statistical Package for the Social Sciences (SPSS) version 22.0, with a significance threshold set at *p* < 0.05 (two-tailed).

## 3 Results

### 3.1 Participants and baseline characteristics

A total of 86 participants were recruited for the study, comprising 40 individuals (17 males and 23 females) in the DED group and 46 individuals (24 males and 22 females) in the control group. Statistical analysis revealed no significant differences in age (*p* = 0.575), sex (*p* = 0.370), or forehead temperature (*p* = 0.076) between the two groups. Significant differences were observed in both the NIBUT (*p* < 0.001) and OSDI (p < 0.001) between the two groups, as detailed in Table 1.

**Table 1:** Demographic and clinical data by group.

Variables	DED group	Control group	*p*-value
Participants/eyes (n)	40/40	46/46	–
Age (year)	50.00 ± 15.88	51.70 ± 11.29	0.575
Female, n (%)	23 (58%)	22 (48%)	0.370
Forehead temperature (°C)	31.76 ± 0.93	31.37 ± 1.02	0.076
NIBUT (s)	5.74 ± 1.36	11.53 ± 4.12	< 0.001*
OSDI	23.14 ± 3.89	10.12 ± 1.85	< 0.001*

NIBUT, non-invasive breakup time; OSDI, Ocular Surface Disease Index. *p < 0.05.

### 3.2 Central corneal temperature in the DED and control groups

**Figure 2:**
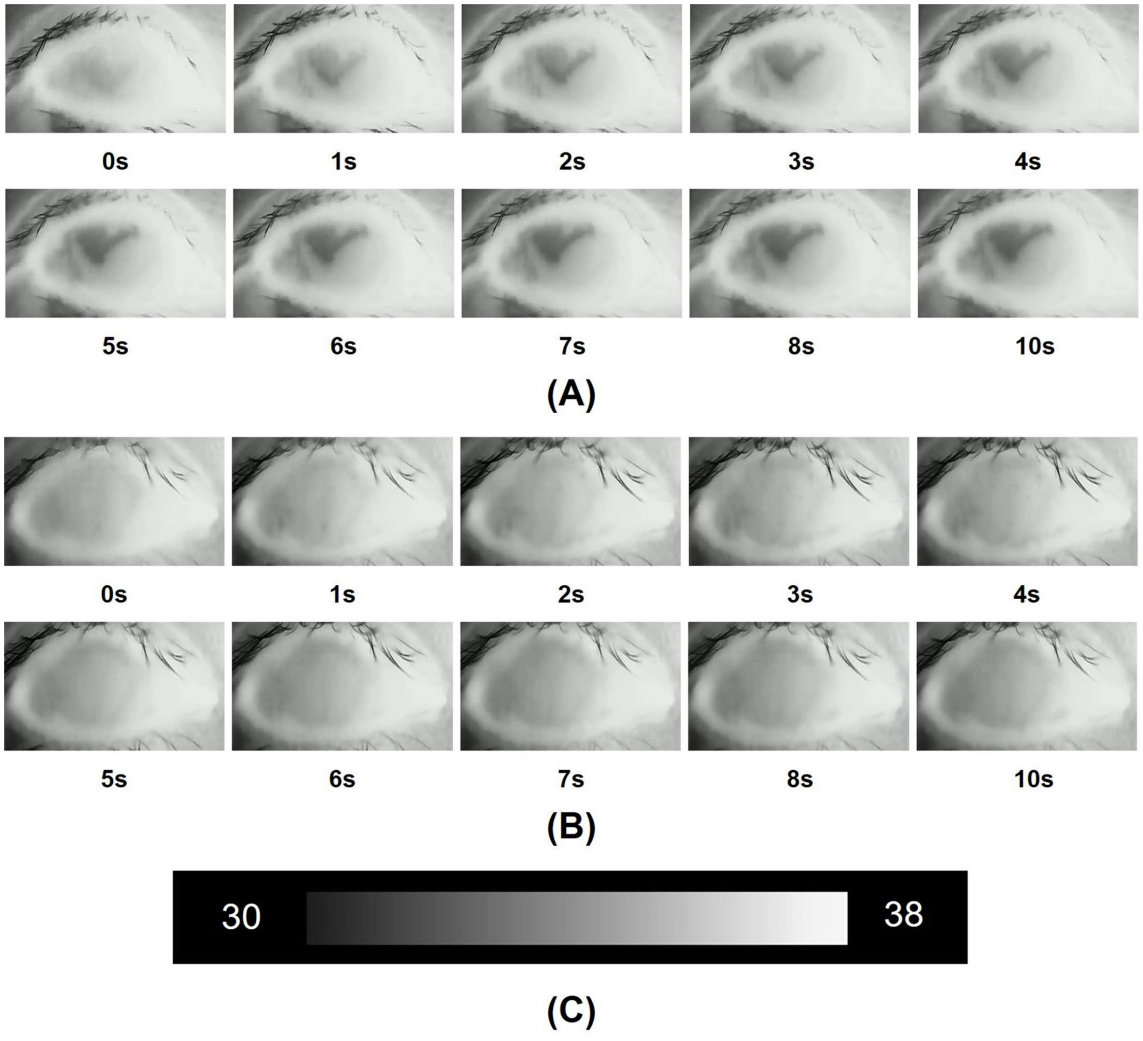
**(A)** Infrared thermal images of the dry eye were obtained over 10 s after the beginning of the measurements. In a dry eye, the ocular surface temperature (OST) gradually changes from a high gray level to a low gray level, signifying a drop in temperature over the 10-s period; **(B)** Infrared thermal images of the normal eye were obtained over 10 s after the beginning of the measurements. In a normal eye, the gray level of the images shows almost no variation, and the temperature stays stable over the 10-s period; **(C)** The temperature scale ranged from 30°C to 38°C.

**Figure 3:**
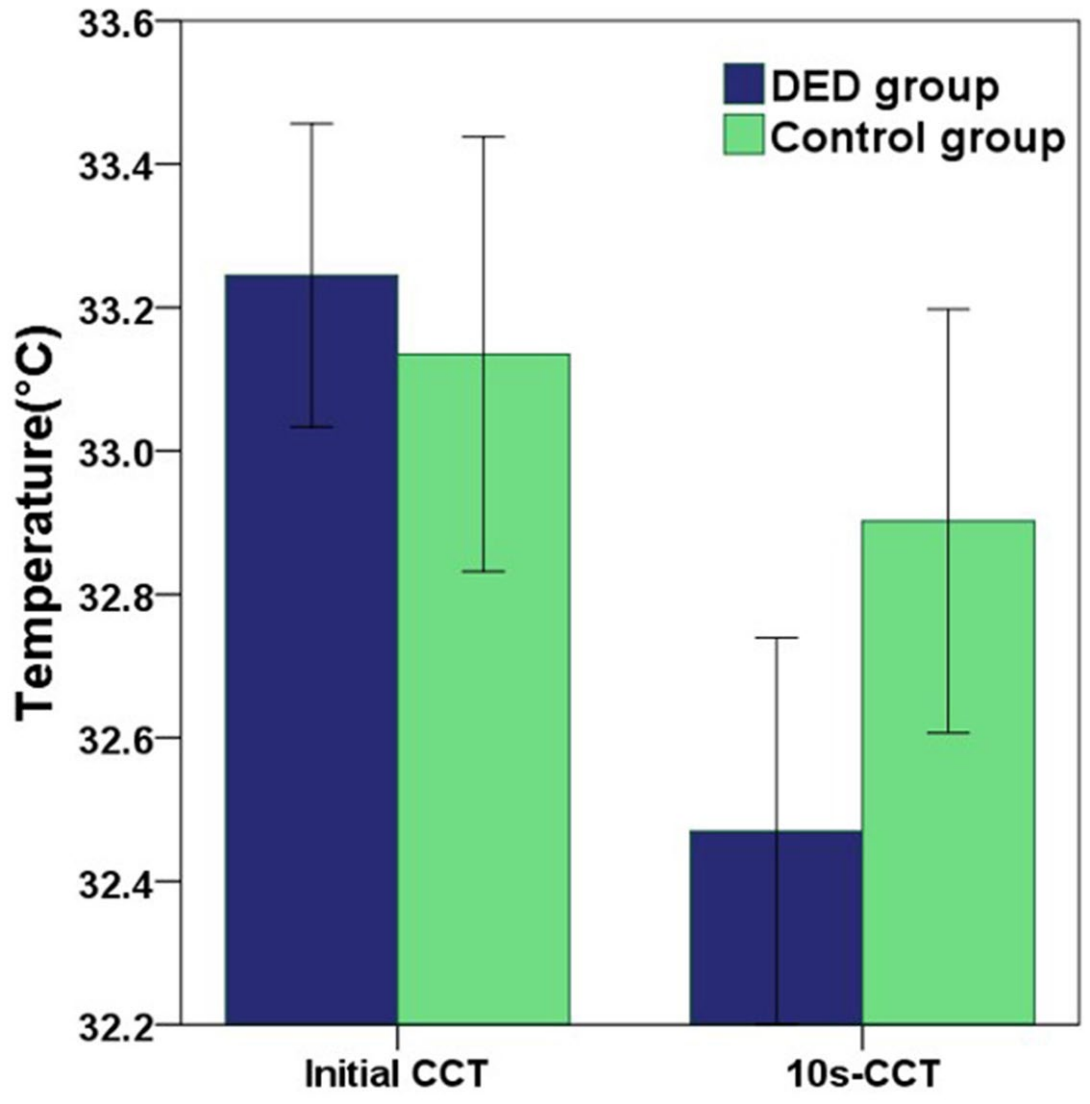
The graph showing the differences in initial CCT and 10s-CCT between the DED and control groups. Error bars = 95% confidence interval. The comparisons of mean values were performed using unpaired t-tests. Compared with the control group, the mean initial CCT in the DED group was greater, but there was no significant difference (*p* = 0.549). However, the mean 10s-CCT in the DED group was significantly lower than that in the control group (*p* = 0.034).

**Figure 4:**
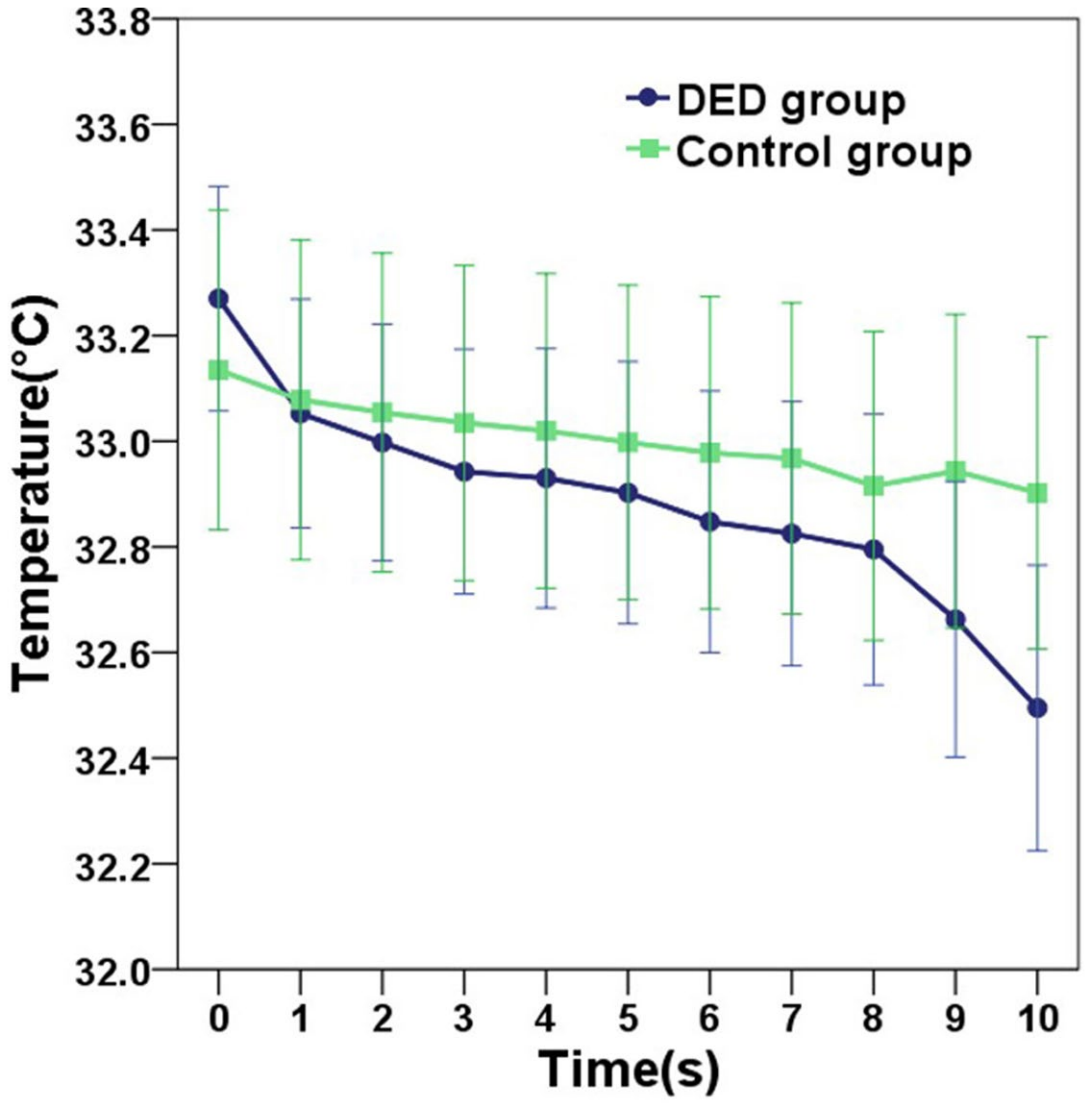
The graph showing the continuous variation in the central corneal temperature (CCT) of the DED and control groups within 10 s. Error bars = 95% confidence interval. Compared with the control group, the mean CCT in the DED group decreased more rapidly over the 10-s period.

Compared with the control group, for participants diagnosed with DED, the mean initial CCT was greater, however, this difference did not reach statistical significance (*p* = 0.549). In contrast, the mean 10s-CCT of participants in the DED group was lower than that of participants in the control group, and there is a significant difference (*p* = 0.034). The change in CCT within 10 s of the DED group occurred faster than that of the control group, and this change was significantly different (*p* < 0.001). The alterations observed in the infrared thermal images of the ocular surface were conspicuous in the DED group (Figure 2A). In contrast, no obvious changes were observed in the control group (Figure 2B). The temperature scale ranges from 30°C to 38°C (Figure 2C). Table 2 and Figure 3 provide a detailed comparison of these findings, whereas Figure 4 illustrates the continuous variation in CCT over the 10 s for both the DED and control groups.

**Table 2:** Central corneal temperature (°C) in the DED and control groups.

CCT	DED group	Control group	*p*-value
Initial CCT (°C)	33.25 ± 0.66	33.14 ± 1.02	0.549
10s-CCT (°C)	32.47 ± 0.84	32.90 ± 0.99	0.034*
Change in CCT within 10 s (°C)	0.78 ± 0.30	0.23 ± 0.20	< 0.001*

CCT, central corneal temperature; Initial CCT, initial central corneal temperature; 10s-CCT, tenth-second central corneal temperature; Change in CCT within 10 s, change in central corneal temperature measured within 10 s. *p < 0.05.

### 3.3 Comparison of the standard deviation of the temperature values within the ROI between the DED and control groups

**Figure 5:**
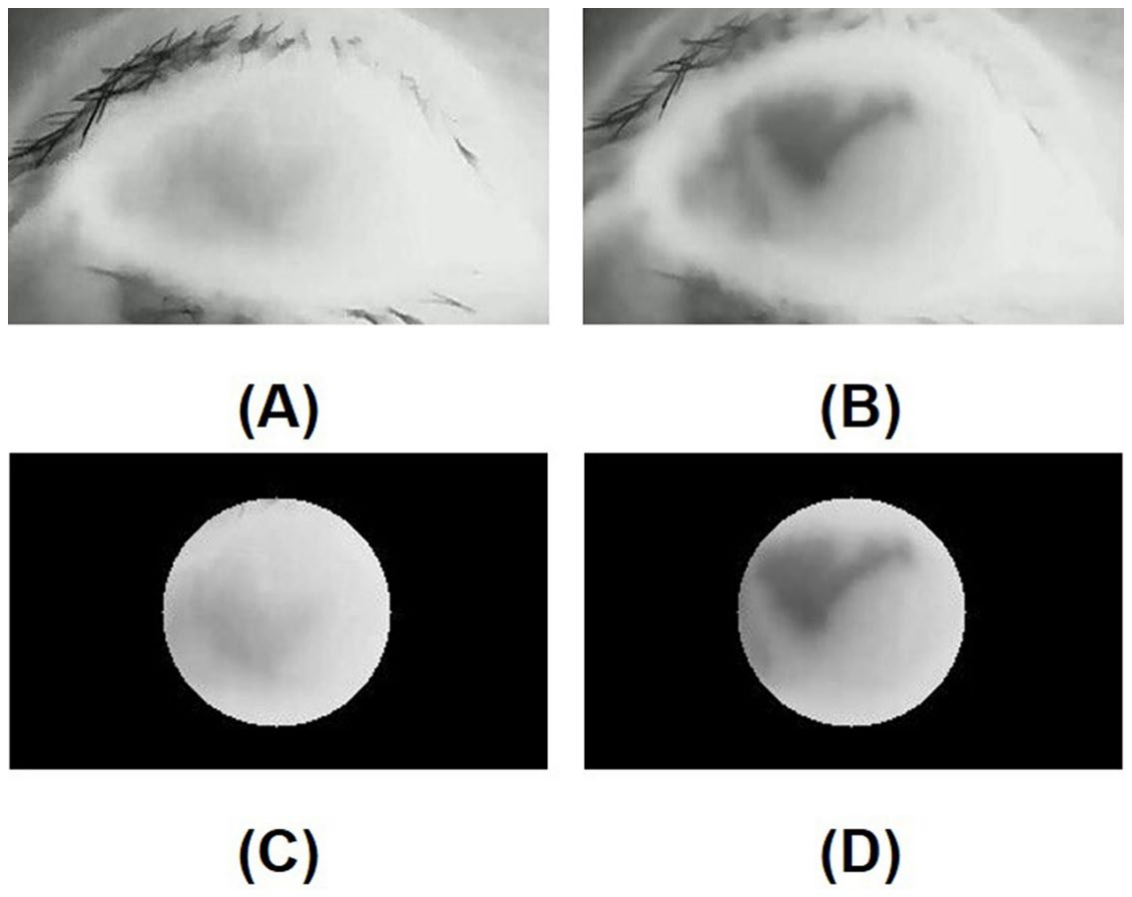
**(A)** Initial infrared thermal image of the dry eye; **(B)** Tenth-second infrared thermal image of the dry eye; **(C)** Region of interest (ROI, 8mmφ) of the initial infrared thermal image on the central cornea of the dry eye; **(D)** Region of interest (ROI, 8mmφ) of tenth-second infrared thermal image on the central cornea of the dry eye; In a dry eye, after opening the eye, a lower temperature area is generated due to the instability of the tear film.

**Figure 6:**
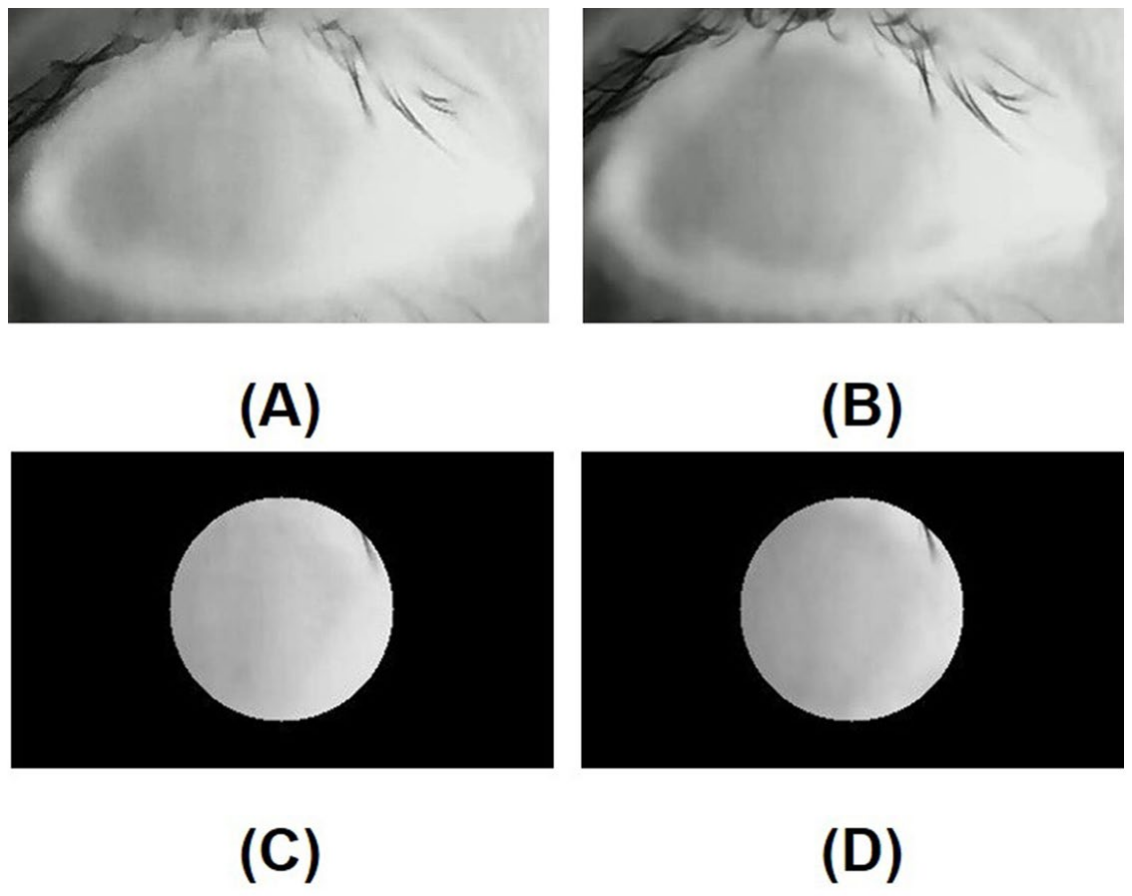
**(A)** Initial infrared thermal image of the normal eye; **(B)** Tenth-second infrared thermal image of the normal eye; **(C)** Region of interest (ROI, 8mmφ) of initial infrared thermal image on the central cornea of the normal eye; **(D)** Region of interest (ROI, 8mmφ) of tenth-second infrared thermal image on the central cornea of the normal eye; In a normal eye, after opening the eye, a lower temperature area is not generated due to the stability of the tear film.

In the DED group, the mean standard deviation of the initial temperature values within the ROI (SD of initial TVs within ROI) was similar to that in the control group (*p* = 0.926). In contrast, the mean standard deviation of the tenth-second temperature values within the ROI (SD of 10s-TVs within ROI) in the DED group was greater than that in the control group, and there is a significant difference (*p* = 0.016). Figures 5A,B are the initial and tenth-second infrared thermal images of dry eyes, respectively. Figures 5C,D are the corresponding ROIs. Figures 6A,B are the initial and tenth-second infrared thermal images of normal eyes, respectively. Figures 6C,D are the corresponding ROIs. A detailed comparison of these findings is shown in Table 3.

**Table 3:** Standard deviation of the temperature values within the ROI between the DED and control groups.

SD of TVs within ROI	DED group	Control group	*p*-value
SD of initial TVs within ROI (°C)	0.23 ± 0.07	0.22 ± 0.05	0.926
SD of 10s-TVs within ROI (°C)	0.44 ± 0.20	0.35 ± 0.15	0.016*

SD of TVs within ROI, standard deviation of the temperature values within the ROI; SD of initial TVs within ROI, standard deviation of the initial temperature values within the ROI; SD of 10s-TVs within ROI, standard deviation of the tenth-second temperature values within the ROI. *p < 0.05.

### 3.4 Comparisons of the change in central corneal temperature before and after tear film break-up in the DED group

**Figure 7:**
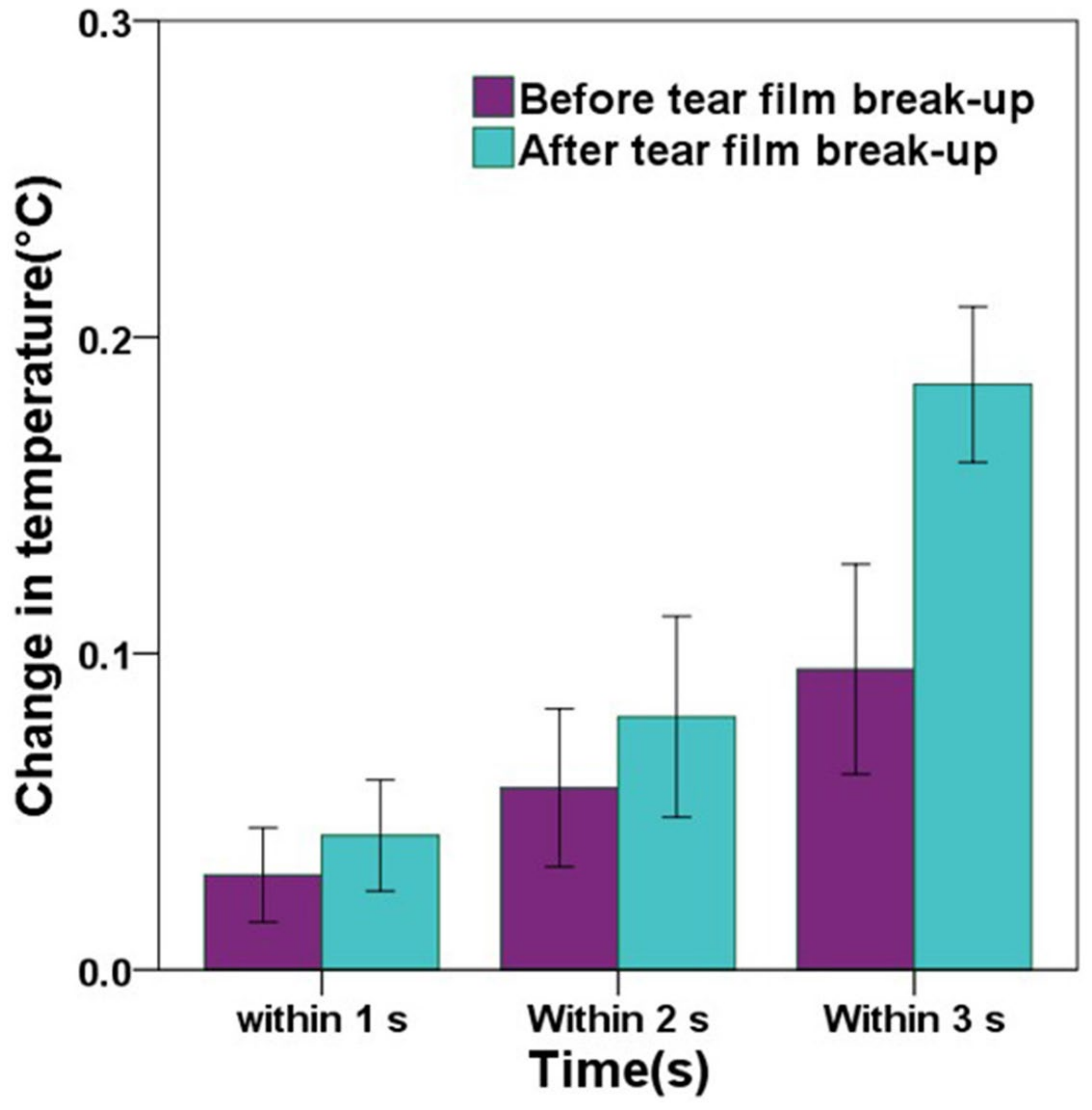
The graph showing the comparisons of the change in central corneal temperature (CCT) before and after tear film break-up in the DED group. Error bars = 95% confidence interval. The comparisons of mean values were performed using paired t-tests. In the DED group, the changes in CCT within 1 s, 2 s, and 3 s after tear film break-up were greater than those before tear film break-up, but only the change in CCT within 3 s before and after tear film break-up has a significant difference (*p* < 0.001).

In the DED group, the mean change in CCT within 1 s after tear film break-up was greater than that before tear film break-up, but there was no significant difference (*p* = 0.281). The mean changes in CCT within 2 s before and after tear film break-up showed the same result (*p* = 0.277). The mean change in CCT within 3 s after tear film break-up was significantly greater than that before tear film break-up, and there was a significant difference (*p* < 0.001). Detailed comparisons can be found in Figure 7 and Table 4.

**Table 4:** Comparisons of the change in central corneal temperature before and after tear film break-up in the DED group.

Time of the change in CCT	Before tear film break-up	After tear film break-up	*p*-value
Change in CCT within 1 s (°C)	0.03 ± 0.05	0.04 ± 0.05	0.281
Change in CCT within 2 s (°C)	0.06 ± 0.08	0.08 ± 0.10	0.277
Change in CCT within 3 s (°C)	0.10 ± 0.10	0.19 ± 0.08	< 0.001*

CCT, central corneal temperature. *p < 0.05.

### 3.5 Differences in the central corneal temperature between males and females

For the participants in the DED group, the mean initial CCT and mean 10s-CCT of males were similar to those of females, with no significant difference (*p* = 0.884, *p* = 0.232, respectively). However, the mean change in CCT within 10 s of females was greater than that of males, and this difference was significant (*p* = 0.001). In the control group, there were no significant differences in initial CCT, 10s-CCT, and change in CCT within 10 s between males and females (*p* = 0.825, *p* = 0.964, and *p* = 0.169, respectively). A comparison of all males and females in both groups showed no significant differences in initial CCT and 10s-CCT (*p* = 0.845, *p* = 0.379, respectively). Although there was also no significant difference in change in CCT within 10 s between males and females in both groups, the mean change in CCT within 10 s of females was greater than that of males. Detailed comparisons are shown in Table 5.

**Table 5:** Differences in the central corneal temperature between males and females.

Group	CCT	Males	Females	*p*-value
DED group	Initial CCT (°C)	33.29 ± 0.59	33.26 ± 0.73	0.884
	10s-CCT (°C)	32.68 ± 0.75	32.36 ± 0.90	0.232
	Change in CCT within 10 s (°C)	0.61 ± 0.23	0.90 ± 0.29	0.001*
Control group	Initial CCT (°C)	33.17 ± 1.22	33.10 ± 0.78	0.825
	10s-CCT (°C)	32.90 ± 1.21	32.91 ± 0.73	0.964
	Change in CCT within 10 s (°C)	0.27 ± 0.21	0.19 ± 0.18	0.169
Both groups	Initial CCT (°C)	33.22 ± 1.00	33.18 ± 0.75	0.845
	10s-CCT (°C)	32.81 ± 1.04	32.63 ± 0.85	0.379
	Change in CCT within 10 s (°C)	0.41 ± 0.27	0.55 ± 0.43	0.066

CCT, central corneal temperature; Initial CCT, initial central corneal temperature; 10s-CCT, tenth-second central corneal temperature; Change in CCT within 10 s, change in central corneal temperature measured within 10 s. *p < 0.05.

## 4 Discussion

Compared with that of normal eyes, the mean initial central corneal temperature (initial CCT) of dry eyes was greater, although there was no significant difference between the two groups. This research result is consistent with the findings of several previous studies (31, 32). The underlying factors contributing to this outcome may be associated with inflammation (33). In dry eyes, there may be inflammatory manifestations on the ocular surface (34, 35), which can potentially result in an elevated central corneal temperature (CCT). Additionally, the thickness of the tear film may be one of the reasons for this result. It has been noted in the literature that infrared radiation within the wavelength range of 8 to 13 μm is absorbed by water, with the extent of absorption being contingent upon the thickness of the water layer (31). Because the tear film is less thick in dry eyes (36, 37), less infrared radiation is absorbed by the tear film, resulting in a higher CCT. This finding may also be related to the stability of the tear film (25, 38). However, some researchers have reported that the initial CCT of dry eyes is lower than that of normal eyes (11, 30). This difference could be attributed to a combination of technical factors, such as the precision of the instruments, the conditions under which the measurements are taken, and the methodologies utilized (18).

Compared with normal eyes, dry eyes presented a significantly faster decrease in CCT. This result is consistent with the results of other studies (11, 12, 25, 30). Due to the rapid decrease in CCT of dry eyes, the mean tenth-second central corneal temperature (10s-CCT) of participants in the DED group was significantly lower than that of participants in the control group (11). As previously reported in the literature, DED is characterized by two primary pathogenic mechanisms: diminished aqueous tear production resulting from lacrimal gland dysfunction and increased evaporation of the tear film (24, 27, 39, 40). Consequently, both the rate of tear secretion and the stability of the tear film may influence the changes in CCT following a blink (25). Mapstone posits that the thermal dynamics associated with the ocular surface can be elucidated through the mechanisms of convection, radiation, and evaporation. Specifically, the movement of air across a surface results in heat dissipation via convection, whereas radiant heat loss transpires when thermal energy is transferred to cooler surrounding temperatures (41). When the eye is open and the tear film evaporates, the ocular surface experiences a decrease in temperature. This cooling effect occurs as a result of the positive latent heat of vaporization, whereby heat is dissipated into the surrounding environment during the transition from liquid to gas (42). Compared with that of normal eyes, the tear film stability of dry eyes was lower, increasing the tear evaporation rate and resulting in a greater change in the CCT.

In this study, the standard deviation of the temperature values within the ROI (SD of TVs within ROI) on the central cornea was assessed to evaluate the uniformity of corneal temperature between the DED group and the control group. The standard deviation of the initial temperature values within the ROI (SD of initial TVs within ROI) of dry eyes was not significantly different from that of normal eyes. However, the mean standard deviation of the tenth-second temperature values within the ROI (SD of 10s-TVs within ROI) in the DED group was significantly greater than that in the control group. When the tear film is abnormal, it affects the rate of evaporation in some areas of the ocular surface. This disrupts the uniformity of the corneal temperature, manifested as an increase in the SD of TVs within ROI. For normal eyes, the tear film is stable, and the variation in CCT is small and smooth during the evaporation process. For dry eyes, the tear film remains relatively intact shortly after opening the eyes and the uniformity of the corneal temperature shows little difference compared to normal eyes. However, as the time with eyes open increases, the instability of the tear film results in a greater degree of variation in the CCT and the generation of a lower temperature area on the tear film. In other words, tear film break-up compromised the uniformity of the corneal temperature (12, 26).

In the DED group, the mean change in CCT within 3 s after tear film break-up was significantly greater than that before tear film break-up, and there was a significant difference. When the eyes are open, the tear film undergoes evaporation, causing it to become thinner and eventually break up. Tear film break-up results in a lower temperature area on the tear film (26). As the area of tear film break-up continues to increase, the decrease in CCT also accelerates.

For the participants in the DED group, the mean change in CCT within 10 s of females was significantly greater than that of males. In previous studies, it has been found that DED is more prevalent in females compared to males (43, 44). The gender differences in corneal temperature in the DED group may help us further understand the pathogenesis of the dry eye, which is a topic worth studying.

This study also has some limitations. Firstly, it does not classify DED, and different types of the DED may exhibit variations in high-resolution ocular thermography, which warrants further investigation. Secondly, although we explored the gender differences in corneal temperature, the sample size was small, and a larger sample study is needed for validation.

According to this study, there are significant differences in CCT between dry eyes and normal eyes. The evaporation of the tear film results in a cooling of the ocular surface (42), so measuring the changes in CCT during the inter-blink period can be used as an index of tear film stability. This index may have greater clinical value for the diagnosis of evaporative dry eyes. At the same time, the uniformity of the corneal temperature can indicate whether there is a tear film break-up. Additionally, doctors can observe high-resolution infrared thermography images and videos to assist in diagnosing dry eyes. Compared with the fluorescein breakup time (FBUT), ocular thermography does not require fluorescein staining and is relatively simple to perform. Compared with the non-invasive tear breakup time (NIBUT), ocular thermography allows for direct observation of tear film changes over time without the Placido disk. These aspects highlight the potential of ocular thermography in the diagnosis of DED. However, the sensitivity of ocular thermography to environmental conditions and the need for precise calibration present challenges for its clinical use.

## Data Availability

The raw data supporting the conclusions of this article will be made available by the authors, without undue reservation.
